# Supplementary material for: Cleared tissue dual-view oblique plane microscopy
Source: Biomed Opt Express. 2025 Aug 22;16(9):3725–39. doi: 10.1364/BOE.572592 (PMC12684026; doi:10.1364/BOE.572592)
Supplement: Supplementary file 1 [file boe-16-9-3725-s001.pdf]

Supplemental Document

Biomedical Optics EXPRESS

Cleared tissue dual-view oblique plane microscopy: supplement

L. DVINSKIKH,[1,2,*] H. SPARKS,[1,3] L. ROWE-BROWN,[1,3] W. HONG,[1] D. ENNIS,[2] R. SARNATARO,[4] D. CARRENO,[3,4] F. S. TISSOT,[3,5,6] C. LO CELSO,[3,5,6] D. T. RIGLAR,[3,7] P. CUNNEA,[2] I. A. MCNEISH,[2] AND C. DUNSBY[1,3]

[1] *Department of Physics, Imperial College London, UK*
[2] *Department of Surgery and Cancer, Imperial College London, UK*
[3] *The Francis Crick Institute, UK*
[4] *Centre for Neural Circuits and Behaviour, University of Oxford, UK*
[5] *Department of Life Sciences, Imperial College London, UK*
[6] *Centre for Haematology, Department of Immunology and Inflammation, Imperial College London, UK*
[7] *Department of Infectious Diseases, Imperial College London, UK*
[*] *liuba.dvinskikh@ic.ac.uk*

This supplement published with Optica Publishing Group on 22 August 2025 by The Authors under the terms of the Creative Commons Attribution 4.0 License in the format provided by the authors and unedited. Further distribution of this work must maintain attribution to the author(s) and the published article's title, journal citation, and DOI.

Supplement DOI: https://doi.org/10.6084/m9.figshare.29908187

Parent Article DOI: https://doi.org/10.1364/BOE.572592

Cleared tissue dual-view oblique plane microscopy: Supplemental Document

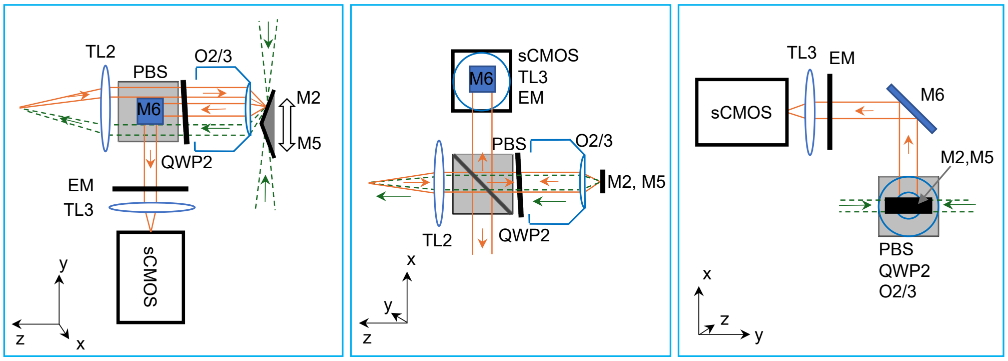

Fig. S1. Three-way view of the polarizing beam splitter and periscope mirror assembly geometry in the remote-refocusing path of the dOPM system. Green dashed lines indicate the excitation light and solid orange lines indicate the emitted fluorescence. For unpolarized fluorescence emission in the case of rapidly tumbling fluorophores, approximately half of the light is transmitted during the first pass through the PBS. For partially polarized fluorescence in the case of fluorophores static on the timescale of the fluorescence lifetime, approximately 75% of the light is transmitted on the first pass (obtained by solving Eq. 10.1 in [1] under the condition of steady state anisotropy r = 0.4). The now linearly polarized light is converted to circularly polarized light by QWP2. Upon reflection off M2 or M5, the handedness of the circularly polarized light is reversed. After passing through QWP2 the light is linearly polarized in the orthogonal direction, reflected by the PBS, and directed via periscope mirror M6 to the camera. For the excitation, the light is circularly polarized upon incidence on M2 and M5, converted to linear by QWP2, with most of the light transmitted through the PBS to the sample.

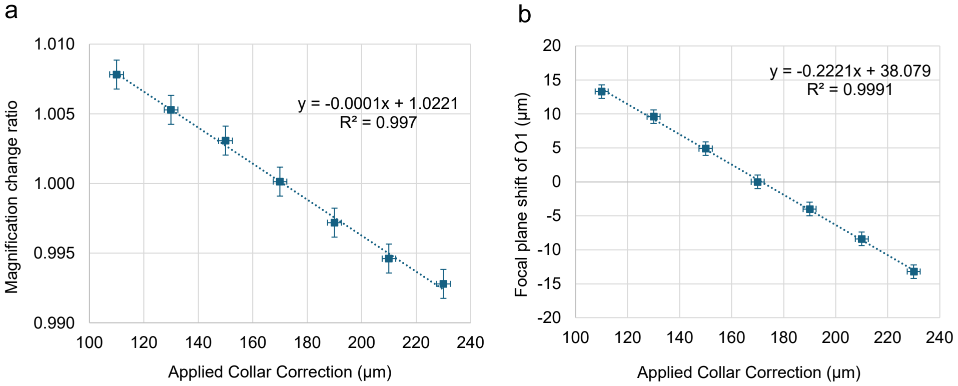

Fig. S2. (a) Change in the lateral magnification (average for x and y) of the first microscope (O1 and TL1) for different correction collar (CC) settings, with the available CC range causing a 1.5% change in the magnification. (b) Required axial translation of O1 to refocus on the sample after changing the correction collar value. Vertical error bars in (a) are the propagated errors based on single pixel dimensions, and in (b) correspond to the accuracy of in-focus readings of the motorized O1 z-drive position. Horizontal error bars correspond to the estimated errors of manual correction collar adjustment.

Sample	Beads	Mouse omentum	Fly brain	Mouse colon	Mouse bone marrow
Medium	TDE	TDE	Vectashield	Ce3D	Ce3D
RI	1.406, 1.45, 1.5	1.406	1.45	1.5	1.46
CC position	0.11-0.23	0.17	0.19	0.21	0.20
Z_{0RF} (μm)	150	100	100	150	150
No of tiles (H × V)	1 × 1	8 × 4	3 × 2	3 × 2	30 × 1
Exposure time (ms)	100	15	20	200	10
Pixel binning	1 × 1	2 × 2	2 × 2	1 × 1	1 × 1
Step size (μm)	1	2	2	1	1 (stage scanned)
Image planes per view	241	101	121	201	2000
Excitation λ (nm)	488	488, 642	488	488, 561, 642	488, 561

Table. S1. Summary of the image acquisition and processing parameters for each one of the samples for the data presented in the main text, including refractive index (RI), correction collar (CC) position, the distance of the plane of zero remote refocus to the coverslip surface, Z_{0RF}, number of horizontal (H) and vertical (V) tiles and their respective overlap, exposure time, pixel binning, step size, total number of image planes per view, and laser excitation wavelength λ.

Note S1: Preparation of RI-tuned fluorescent bead samples

The RI-tuned fluorescent bead phantoms were generated by mixing 2% agarose in liquid form with 2,2'-Thiodiethanol (TDE, Sigma Aldrich) to obtain the desired refractive index (1.406, 1.45 and 1.5), checked using a refractometer (Abbe 5 Refractometer, Bellingham and Stanley). Further agarose powder was added to allow the liquid to set into a gel when cooled, adjusting the final agarose concentration to ~2.2%, with the RI re-measured and adjusted through addition of TDE if necessary. Fluorescent beads with 170 nm actual diameter (Tetraspeck, Invitrogen T7280) were added to the agarose (heated to form a liquid) at 1:40 concentration, and after careful mixing, pipetted into a #1.5H glass bottom 8-well μ-slide (Ibidi, 80807).

Note S2: Preparation of mouse ovarian cancer omentum samples

The experiment was carried out under approval of the local Ethical Review Committee according to UK Home Office guidelines (PPL PA780D61A). ID8-F3 mouse omental tumours were fixed in 10% neutral buffered formalin for at least 24 hours, then placed in 70% ethanol before embedding in 5% low gelling temperature agarose (Thermo Scientific) and macro-sectioned into 250 μm slices on a Leica VT1200S vibratome. The slices were washed and resuspended in phosphate-buffered saline (PBS) and labelled with the DNA stain DRAQ5 (Biostatus) at 10 μM concentration for 30 minutes. After further washes and resuspension in PBS, the samples were cleared and RI-matched through sequential incubation in TDE: 20% TDE/PBS for 2 hours in 37 degrees on a rocker, followed by 36.5% TDE/PBS (n = 1.406) for 2 hours in 37 degrees on a rocker, and left on rocker at room temperature overnight. The samples were mounted in the second TDE dilution solution onto a microscope slide, using a gene frame to create a volume, and sealed with a #1.5 coverslip.

Note S3: Preparation of fly brain samples

A detailed account of the fly brain sample preparation is provided in Sarnataro et al. [2]. Briefly, *Drosophila melanogaster* fruit flies expressing the mitochondria-localised fluorophore GFP (RRID: BDSC_8442) under the control of the driver line *R23E10-GAL4* (RRID: BDSC_49032) were reared on medium containing yeast, cornmeal, molasses, and agar under a 12 h light:12 h dark cycle at 25 °C with 60% relative humidity. Brains of randomly selected females were dissected with sharp forceps 4-6 days post eclosion in PBS, quickly transferred to Eppendorf tubes on ice, and fixed in 4% (w/v) paraformaldehyde (Electron Microscopy

Sciences) diluted in 0.3% (v/v) TritonX-100 in PBS (PBST) for 20 min on a nutator. Brains were quickly washed twice and then three times rocking for 20 min each, with PBST, and mounted in Vectashield antifade mounting medium (Vector Laboratories) on a microscopy slide (Fisherbrand), covered with a #1,5 coverslip (VWR), and sealed with nail polish.

Note S4: Preparation of Ce3D-cleared colon tissue.

A frozen mouse colon sample was obtained from an experiment described in Carreno et al. [3], carried out under approval of the local Ethical Review Committee at Imperial College London according to UK Home Office guidelines (PPL PP7088487). Briefly, mice received an engineered commensal E. coli expressing constitutive fluorescent mKate2 and inducible mVenus to study gut inflammation. Dissected and fixed mouse colon was cryosectioned using a Leica Cryostat to remove the first 200 μm and expose the lumen for more efficient antibody permeability. Immunolabelling was performed using a primary Chicken IgY anti-GFP (Abcam) and Donkey anti-chicken IgY (H+L), CF™ 488A (Sigma-Aldrich) secondary antibodies for immunolabelling of mVenus; Goat IgG anti-RFP (Cambridge Biosciences) primary and Alexa Fluor 568 donkey anti-goat IgG (H+L) (Abcam) secondary antibodies for immunolabelling of mKate2; and Ulex Europaeus Agglutinin I (UEA I), DyLight® 649 (Vector Laboratories) preconjugated marker of the mucin layer. Ce3D clearing of mouse colon was adapted from Li et al. [4] as described in Carreno et al. [3]. The sample was placed in freshly made Ce3D clearing solution, protected from light in a cryomold for 10 days, replacing the Ce3D solution every 48h. Optically cleared mouse colon was mounted onto a microscope slide using gene frames to create a volume, Ce3D clearing solution as a mounting media (n = 1.5) and sealed with a glass cover slip with #1.5 thickness.

Note S5: Preparation of Ce3D-cleared mouse bone marrow tissue.

All animal work was in accordance with the animal ethics committee (AWERB) at Imperial College London, UK and UK Home Office regulation (ASPA, 1986). All mice were bred and housed at Imperial College London or Sir Francis Crick Institute (PP9504146). C57BL/6 WT mice were purchased from Charles River (United Kingdom). Female mice > 6 weeks old were used. Animals were housed in Tecniplast mouse greenline cages with appropriate bedding and enrichment. The temperature, humidity and light cycles were kept within the UK Home Office code of practice, with standard diet and water *ad libitum*, the temperature between 20 and 24 °C, the room humidity at 45–65% and a 12-hours light /12-hours dark cycle with a 30-min dawn and dusk period to provide a gradual change.

Samples preparation and immunostaining were performed as previously described in [5]. Briefly, bones (femur) were harvested and fixed in 4% formaldehyde at 4°C. Bones were decalcified for 10 days in 10 % EDTA and embedded in 4 % low EEO agarose (Sigma, A0169) and sectioned at 250 μm thickness s using a Leica T1000 Vibratome. All the following steps were performed under agitation at room temperature. Sections were incubated in 20% CUBIC-1 reagent (urea (25 wt% final concentration), Quadrol (25 wt% final concentration), Triton X-100 (15 wt% final concentration) in dH_2O) [6] for 48 hours and rinsed in Tris-buffered saline (TBS). Unspecific antigen binding was avoided using blocking buffer solution (TBS 0.1% Triton, 10% DMSO, 5% normal donkey serum) overnight following incubation with primary antibodies rat CD45R (B220) 14-0452-82 eBioscience™ (Thermofisher) and rabbit Ly-6C PA5-119794 (ThermoFischer), diluted in blocking buffer for 48 hours. Bone marrow sections were then incubated with secondary antibodies (Donkey anti-Rat IgG (H+L) Highly Cross-Adsorbed Secondary Antibody, Alexa Fluor™ 488 A-21208 (ThermoFischer) and Donkey anti-Rabbit IgG (H+L) Highly Cross-Adsorbed Secondary Antibody, Alexa Fluor™ 594 (ThermoFischer) for 48 hours. The bone marrow section were mounted in the Ce3D clearing solution [7] Methlyacetamide 40% (Sigma) diluted in TBS, 1.455g histodenz (sigma) per 1ml 40% Methlyacetamide, 4% DABCO (Sigma)) using silicon isolator (ThermoFischer P18175) on Superfrost PlusTM slides.

RI	CC (µm)	No. of Beads	X-FWHM (µm)		Y-FWHM (µm)		Z-FWHM (µm)		Z*-FWHM (µm)	
			Median	IQR	Median	IQR	Median	IQR	Median	IQR
1.4	110	4388	0.65	0.24	0.69	0.16	1.90	0.32	3.90	0.61
	130	4371	0.55	0.14	0.65	0.14	1.70	0.28	3.85	0.65
	150	4360	0.50	0.12	0.63	0.14	1.37	0.24	3.79	0.80
	170	4316	0.49	0.11	0.60	0.10	1.23	0.16	3.75	0.79
	190	4368	0.52	0.12	0.64	0.13	1.30	0.20	3.75	0.71
	210	4370	0.56	0.15	0.71	0.16	1.58	0.22	3.74	0.58
	230	4264	0.62	0.19	0.78	0.22	1.97	0.38	3.77	0.51
1.45	110	3324	0.78	0.44	0.74	0.21	2.38	0.64	4.15	0.51
	130	3961	0.65	0.35	0.73	0.19	2.25	0.61	4.02	0.52
	150	4454	0.57	0.26	0.69	0.19	2.05	0.56	3.91	0.56
	170	4447	0.52	0.21	0.68	0.17	1.89	0.63	3.82	0.65
	190	4637	0.51	0.19	0.67	0.15	1.69	0.67	3.76	0.73
	210	4642	0.52	0.18	0.66	0.16	1.46	0.54	3.72	0.76
	230	4558	0.52	0.15	0.67	0.19	1.36	0.44	3.73	0.79
1.5	110	894	0.81	0.43	0.80	0.19	2.56	0.77	4.36	0.58
	130	1074	0.72	0.42	0.74	0.16	2.35	0.77	4.24	0.46
	150	1426	0.62	0.34	0.73	0.15	2.21	0.86	4.11	0.48
	170	1726	0.57	0.28	0.73	0.16	2.08	0.95	4.02	0.51
	190	1992	0.56	0.24	0.72	0.18	1.97	1.05	3.94	0.57
	210	2304	0.55	0.24	0.72	0.20	1.86	1.06	3.87	0.67
	230	2528	0.55	0.23	0.71	0.21	1.79	1.06	3.81	0.69

Table S2: Measured experimental lateral (X,Y), axial (Z) bead FWHM and optical sectioning strength (Z*) for 1.4 (top), 1.45 (middle) and 1.5 (bottom) refractive index (RI) for varying correction collar (CC) positions, calculated as medians and corresponding interquartile ranges for beads across the whole overlapping volume region.

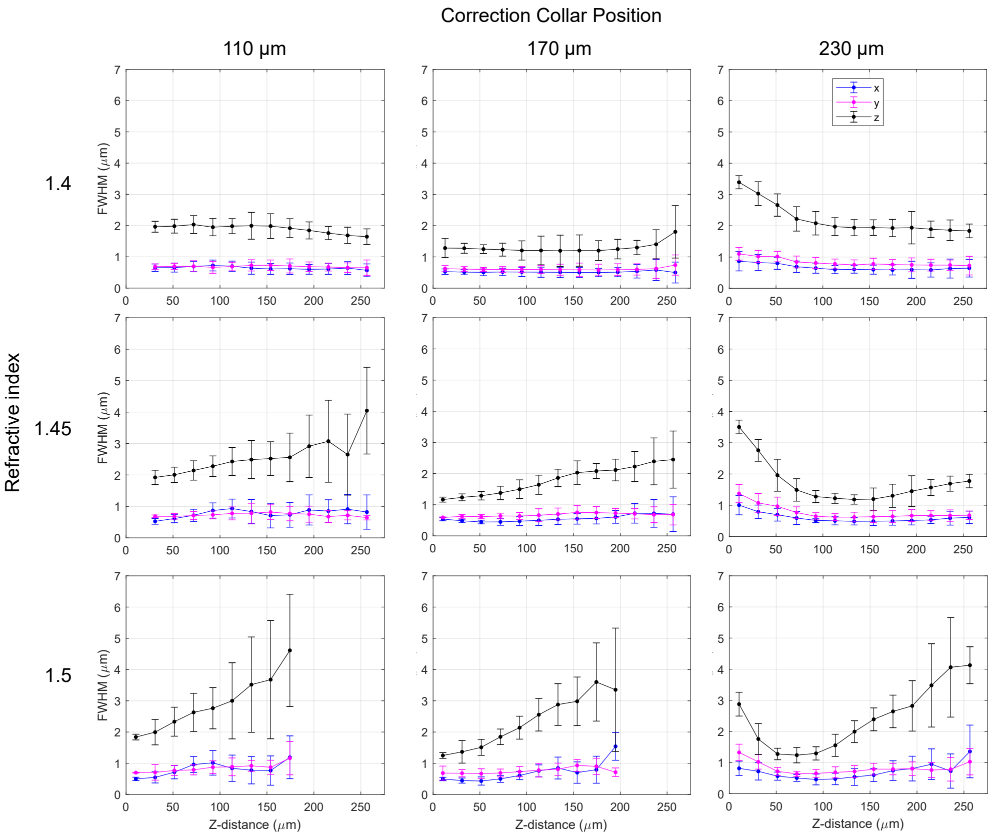

Figure S3: Change in the average raw bead image X-, Y- and Z- bead FWHM (blue, magenta and black respectively), with increasing imaging depth from the coverslip into the sample at various bead sample refractive indices (rows) and different correction collar positions (columns). Error bars represent the standard deviation. Varying offset of the initial data point for each series is due to shifted focus. For this data, the distance from the coverslip to the zero remote refocus plane was ~150 µm.

References

1. J. R. Lakowicz, ed., *Principles of Fluorescence Spectroscopy* (Springer US, 2006).
2. R. Sarnataro, C. D. Velasco, N. Monaco, A. Kempf, and G. Miesenböck, "Mitochondrial origins of the pressure to sleep," Nature 1–7 (2025).
3. D. Carreño, C. M. Robinson, R. Jackson, P. Li, V. Nunes, S. A. Palma-Duran, E. Nye, J. I. MacRae, and D. T. Riglar, "Imaging of live bacterial whole-cell biosensors illuminates spatial sialic acid availability within the inflamed mammalian gut," 2024.10.23.619804 (2024).
4. W. Li, R. N. Germain, and M. Y. Gerner, "High-dimensional cell-level analysis of tissues with Ce3D multiplex volume imaging," Nat Protoc **14**(6), 1708–1733 (2019).
5. G. Adams, F. S. Tissot, C. Liu, C. Brunsdon, K. R. Duffy, and C. L. Celso, "PACESS: Practical AI-based Cell Extraction and Spatial Statistics for large 3D bone marrow tissue images," 2022.12.29.521787 (2024).
6. E. A. Susaki, K. Tainaka, D. Perrin, H. Yukinaga, A. Kuno, and H. R. Ueda, "Advanced CUBIC protocols for whole-brain and whole-body clearing and imaging," Nat Protoc **10**(11), 1709–1727 (2015).
7. W. Li, R. N. Germain, and M. Y. Gerner, "Multiplex, quantitative cellular analysis in large tissue volumes with clearing-enhanced 3D microscopy (Ce3D)," Proceedings of the National Academy of Sciences **114**(35), E7321–E7330 (2017).
